# One-Pot Synthesis of Rubber Seed Shell-Derived N-Doped Ultramicroporous Carbons for Efficient CO_2_ Adsorption

**DOI:** 10.3390/nano12111889

**Published:** 2022-05-31

**Authors:** Xiaoxia Zhang, Meng Rong, Hui Cao, Tianwei Tan

**Affiliations:** 1 Beijing Key Laboratory of Bioprocess, College of Life Science and Technology, Beijing University of Chemical Technology, Beijing 100029, China; zhangxiaoxia0416@163.com; 2 CAS Key Laboratory of Green Process and Engineering, Institute of Process Engineering, Chinese Academy of Sciences, Beijing 100190, China; mrong@ipe.ac.cn

**Keywords:** rubber seed shell, melamine, N-doped porous carbon, ultramicroporosity, CO_2_ adsorption

## Abstract

In this work, a series of novel rubber seed shell-derived N-doped ultramicroporous carbons (NPCs) were prepared by one-step high-temperature activation (500–1000 °C), using melamine as the nitrogen source and KOH as the activator. The effects of the melamine dosage and the activation temperatures on the surface chemical properties (doped N contents and N species), textural properties (surface area, pore structure, and microporosity), CO_2_ adsorption capacities, and CO_2_/N_2_ selectivity were thoroughly investigated and characterized. These as-prepared NPCs demonstrate controllable BET surface areas (398–2163 m^2^/g), ultramicroporosity, and doped nitrogen contents (0.82–7.52 wt%). It was found that the ultramicroporosity and the doped nitrogens significantly affected the CO_2_ adsorption and the separation performance at low pressure. Among the NPCs, highly microporous NPC-600-4 demonstrates the largest CO_2_ adsorption capacity of 5.81 mmol/g (273 K, 1.0 bar) and 3.82 mmol/g (298 K, 1.0 bar), as well as a high CO_2_/N_2_ selectivity of 36.6, surpassing a lot of reported biomass-based porous carbons. In addition, NPC-600-4 also shows excellent thermal stability and recycle performance, indicating the competitive application potential in practical CO_2_ capture. This work also presents a facile one-pot synthesis method to prepare high-performance biomass-based NPCs.

## 1. Introduction

With the rapid development of global industrialization and frequent human activity, excessive CO_2_ has been emitted into the atmosphere, causing the ever-increasing atmospheric CO_2_ concentration and triggering worsening global warming, the melting of polar ice, the rise of the sea level, and serious natural disasters [1,2,3,4]. Thus, carbon capture and sequestration (CCS) have been proposed and regarded as an effective tool to mitigate global CO_2_ emissions. Currently, the mainstream CO_2_ capture technologies mainly include chemical amine absorption, membrane separation, and adsorption [5,6]. Among these technologies, adsorption via solid porous adsorbents has become a promising solution and a research hotspot due to the merits of a reduced regeneration energy penalty, the freedom from corrosion, the easy operation, and the low cost.

So far, various solid CO_2_ adsorbents have been designed and exploited, such as metal organic frameworks MOFs [7], zeolites [8], mesoporous silica [9], covalent organic framework COFs [10], porous organic polymers POPs [11], and N-doped porous carbons NPCs [12]. Remarkably, N-doped porous carbons stand out due to their low-cost preparation, high specific surface area, excellent thermal and chemical properties, designable pore structure, and easy surface functionalization [13].

Typically, N-doped porous carbons are obtained from a two-step chemical activation process: the carbonization of precursors and further chemical activation in the presence of activators (KOH, K_2_CO_3_, KHCO_3_, NaOH, and ZnCl_2_) and nitrogen sources (NH_3_, melamine, and urea) [13]. During high-temperature pyrolysis, the activators will decompose into corrosive intermediates to etch the carbon framework and generate abundant micropores and mesopores [14]. In addition, N function groups can be directly converted to different N species (amine, pyrrole N, pyridine/pyridone N, quaternary nitrogen, and pyridine-N-oxide) and incorporated into the carbon skeleton [15]. These polar N sites on the walls of pores can improve Lewis acid–base interaction between CO_2_ molecules and carbons, consequently enhancing CO_2_ adsorption and selectivity [16].

Up to now, a variety of precursors have been adopted, such as coal, petroleum pitch, polymer, MOFs, and biomass [13]. Of these, biomass resources are green, renewable, and widely available all over the world. Hence, sustainable biomass resources have been widely utilized to prepare N-doped CO_2_ adsorbents in recent decades, including rice husk [12], hazelnut shell [17], water caltrop [18], lotus stalks [19], vine shoots [20], tobacco stems [21], etc. These reported biomass-based NPCs have demonstrated high surface areas and large CO_2_ adsorption capacities (up to 7.42 mmol/g, 273 K/ 1.0 bar) [22], whereas the corresponding CO_2_/N_2_ selectivities are less than 20 [17,23,24]. Notably, the CO_2_ concentration in post-combustion flue gas is relatively low (10–15%, 1 bar) [25]; high CO_2_ selectivity is crucial for practical carbon capture application. The CO_2_ adsorption selectivity of those reported NPCs needs to be further improved for superior CO_2_ capture from dilute gas mixtures.

On the other hand, the common two-step carbonization-activation process is time-consuming and energy-intensive, resulting in very high costs. Recently, the one-pot synthesis of N-doped porous carbons was proposed and received great attention [24]. The carbon precursor, activator, and nitrogen source are fully mixed and activated to produce excellent NPCs at high temperatures. From the point of view of practical large-scale CO_2_ capture application, facile preparation methods to prepare high-performance NPCs are essential. Previous studies have also revealed that ultramicropore (<0.7 nm) and polar heteroatom sites are key to improving CO_2_ affinity over other gases (N_2_, CH_4_) [13]. The activation temperature, activator, and activation process can significantly affect the microporosity and N doping of the resultant NPCs and consequently determine the CO_2_ adsorption performance [26]. However, for one-step direct activation to prepare NPCs, relevant studies on the relationships between activation conditions and porosity and CO_2_ adsorption performance are still limited in the literature.

Rubber crops are traditional, economical agriculture plants in China and Asian countries. In 2016, the estimated rubber seed outputs of China and some Asian countries (Indonesia, Thailand, Malaysia, Vietnam, India, and the Philippines) reach over 2.45 million tons/year [27]. Rubber seed consists of 65% kernel and 35% shell; rubber seed shell (RSS) is a primary byproduct of rubber seed and is regarded as agriculture waste [27]. RSS is a good carbon precursor, and there is growing interest in converting RSS into functional porous carbons. In fact, there are some previous reports available in the literature concerning the preparation of porous carbons from rubber seed shell via physical activation and chemical activation [28,29,30,31,32]. However, to the best of our knowledge, there are still no reports on the preparation of N-doped RSS-derived porous carbons for CO_2_ adsorption. In addition, the surface areas, porosity, and CO_2_ adsorption performance of these reported RSS-based PCs are very limited and need to be significantly improved and studied [29].

Herein, a series of novel N-doped ultramicroporous carbons were prepared via one-pot activation at high temperatures, using rubber seed shell as a precursor, KOH as the activator, and melamine as the nitrogen source. The effects of the nitrogen source dosage and activation temperature (500–1000 °C) on the surface chemical properties, textural properties (surface area, pore structure, pore volume), and CO_2_ adsorption/separation performances were systematically investigated using various characterizations. This study expects to obtain high-performance RSS-based NPCs and to elucidate the relationships between CO_2_ adsorption capacity/selectivity, pore structures, and pore surface chemistry.

## 2. Experimental

### 2.1. Materials and Pretreatment

Rubber seed shell was obtained from Huakun Biotechnology Co., Ltd. (Xishuangbanna, China). The fresh rubber seed shell was washed and then dried in an oven at 80 °C until the water content was less than 3 wt%. The dried rubber seed shell was pulverized with a pulverizer and passed through a 60-mesh sieve to obtain a raw material of rubber seed shell powder, which was used for later use. Melamine (99%), potassium hydroxide (KOH, AR), and hydrogen chloride (HCl, AR) were purchased from Beijing Chemical Works (Beijing, China). Other solvents and reagents were used as received.

### 2.2. Preparation of RSS-Derived NPCs

The pretreated powdered RSS was chemically activated using a mixture of KOH and melamine at high temperatures. The influence of nitrogen source addition was investigated first. The RSS (3.0 g) was thoroughly mixed with KOH (6.0 g) and a varied dosage of melamine powder (melamine/RSS weight ratio = 0.2, 0.4, 0.6, 0.8, 1) in a mortar. Then, the mixture was placed in a porcelain crucible and subjected to a high temperature at 700 °C (5 °C/min) in a tube furnace under nitrogen flow (50 mL/min) and held at the desired temperature for 60 min. Additionally, the obtained products were denoted as NPC-700-x (x = 1~5). After the carbonization was completed, the tube furnace was cooled to room temperature naturally. The obtained carbonized samples were soaked in hydrochloric acid aqueous solution (1 mol/L) for 6 h to remove excessive inorganic salt residue, filtered, and washed repeatedly with deionized water until the pH was neutral. The products were further dried in an oven at 80 °C for 12 h under high vacuum. In addition, the effect of the activation temperatures (500 °C, 600 °C, 800 °C, 900 °C, 1000 °C) was also investigated at the optimized melamine addition (NPC-700-4), and the obtained products were denoted as NPC-y-4 (y = 500~1000).

### 2.3. Instrumentation

Fourier transform infrared spectra (FT-IR, Transmission mode, 400–4000 cm^−1^) of the NPCs were measured on a Thermo Nicolet 8700 (Thermo Fisher, Waltham, MA, USA) by compressing the mixture of samples and KBr into a disk. Additionally, the mass ratio of a sample to KBr was controlled to be 1: 100. Powder X-ray diffraction patterns (PXRD) of NPCs were recorded on a UItima IV diffractometer (Rigaku Corporation, Matsubara-cho. Akishima-shi, Tokyo, Japan) with Cu Kα at 40 kV and 30 mA. Field emission scanning electron microscope (FE-SEM) of the samples was observed on a ZEISS Gemini 300 (Carl Zeiss Microscopy GmbH, Oberkochen, Germany) operated at 10 kV. CHNS elemental analysis was determined by Vario EL cube (Elementar Analysensysteme GmbH, Langenselbold, Germany). X-ray photoelectron spectroscopy (XPS) of the samples was measured on an ESCALAB 250Xi spectrometer (Thermo Fisher, Waltham, MA, USA). The 77 K N_2_ adsorption and adsorption isotherms were measured using an Autosorb-iq gas sorption analyzer (Quantachrom, Boynton Beach Station, FL, USA). All the samples were degassed at 120 °C for 12 h under high vacuum prior to the gas adsorption measurement. The specific surface area, pore size and micropore volume, and pore volume of NPCs were calculated from the obtained 77 K N_2_ adsorption isotherms via different models and conditions.

### 2.4. Gas Adsorption Tests

The static adsorption and desorption isotherms of N_2_ and CO_2_ were measured by using an Autosorb-iq gas sorption analyzer. The CO_2_ (99.999%) and N_2_ (99.999%) gases were utilized for the adsorption and desorption measurements. The adsorption and desorption isotherms at 273 K were measured in an ice-water bath, and isotherms at 298 K were measured in a circulating water bath.

## 3. Results and Discussion

### 3.1. Chemical Structures and Morphology

**Scheme 1:**
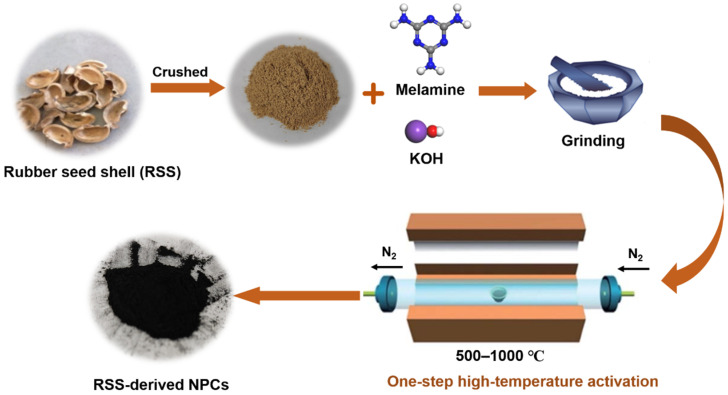
Synthetic illustration of the synthesis of RSS-based NPCs.

**Figure 1:**
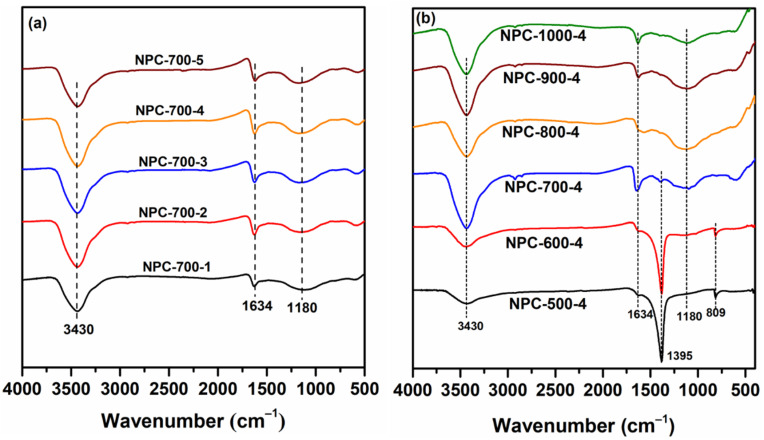
FT-IR spectra of NPC-700-x (**a**) and NPC-y-4 (**b**).

The rubber seed shell was converted to black carbon via one-step activation at a high temperature (500–1000 °C) for 1 h (displayed in Scheme 1). The chemical compositions and surface chemical properties of the NPCs were investigated by FTIR, elemental analysis and XPS. Figure 1 displays the FTIR spectra of NPC-700-x and NPC-y-4. For all the NPC-700-x and NPC-y-4 (y ≥ 700 °C) samples, their FTIR spectra are similar. The absorption band at 1180 cm^−1^ can be attributed to the stretching vibration of C-N [33]. The bands at 3430 cm^−1^ and 1634 cm^−1^ can be associated with both the stretching vibration and the bending vibration of -OH (hydroxyl, carboxyl) and -NH_x_ (amino group, amide) [33,34,35]. It should be noted that NPC-500-4 and NPC-600-4 demonstrate obviously different absorption peaks at 1395 cm^−1^ and 809 cm^−1^. The band at 809 cm^−1^ is a characteristic out-of-plane ring bending of the triazine ring [36]. Additionally, the sharp absorption peak at 1395 cm^−1^ should be related to the stretching vibration of the melem unit [37,38]. It can be reasonably inferred that melamine gradually decomposes into NH_3_, melem, and graphite-like carbon nitrides. These NH_2_-containing intermediates will further react with KOH-related intermediates, carboxyl, hydroxyl, or carbonyl of the RSS precursor. At a lower activation temperature (500, 600 °C), these NH_2_-containing groups may be well incorporated in the carbon skeleton. However, these NH_2_-containing groups will convert to other N-containing groups such as pyridine, pyrrole, and graphitic N under very high activation temperature (>700 °C) [39]. In addition, this is also supported by the gradually weakened absorption band at 1395 cm^−1^ with the activation temperature increase.

**Figure 2:**
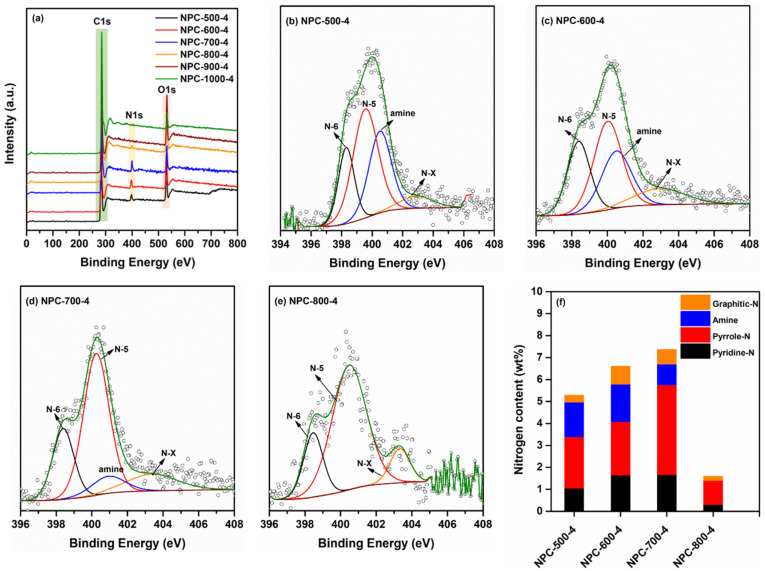
XPS (**a**), N1s XPS (**b**–**e**) spectra of NPC-y-4, and the contents of the nitrogen group in NPC-y-4 (**f**).

The obtained XPS spectra of NPC-700-x and NPC-y-4 are shown in Figure S1 and Figure 2a. It is easily observed that the N1s XPS signal increases with the increasing melamine addition and drops with the increasing activation temperature. Combing the above FTIR spectra analysis of the NPCs, it can be concluded that the doped N content and the N species in the NPCs should be different. Additionally, it is also confirmed by the elemental analysis data and the XPS-derived elemental analysis data (Table 1). From the data shown in Table 1, the C and N contents obtained from the two methods are basically consistent. After high-temperature N-doped activation, the C and N contents of the NPCs were significantly increased, and the O content was greatly reduced. As expected, the doped N contents in the NPC-700-x samples increased with the enhancing melamine addition and the maximum value reached 7.52 wt%. However, the doped N content data of the NPC-y-4 samples show that the activation temperature gradually improved the doped N contents in the range of 500~700 °C (Figure 2a). As the activation temperatures increased to 1000 °C, the doped N contents sharply decreased to 0.82 wt%, suggesting that high activation temperatures will cause the loss of nitrogens during the pyrolysis [15].

**Figure 3:**
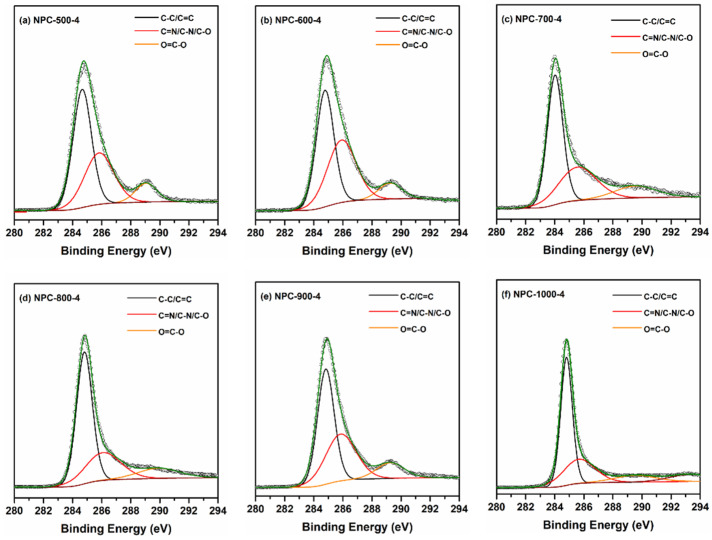
C1s XPS spectra of NPC-500-4 (**a**), NPC-600-4 (**b**), NPC-700-4 (**c**), NPC-800-4 (**d**), NPC-900-4 (**e**) and NPC-500-4 (**f**).

To figure out the N species of the as-prepared N-doped carbons, the N1s XPS spectra are analyzed and shown in Figure 2b–e, Figures S1 and S2b–f. The N1s spectra of four peaks at 398.4, 400.2, 400.6, and 402.8 eV were attributed to pyridine/triazine nitrogen (N-6), pyrrole nitrogen (N-5), amine (-NH_x_), and graphitic nitrogen (N-Q), respectively [13,15,39]. All the NPC-700-x samples show similar N1s spectra and N species. As can be observed from Figure 2f, the N species vary significantly among the NPC-y-4. With the increasing activation temperature (500–700 °C), amine decreased and pyrrolic-N and graphitic-N increased. As the activation temperature reached over 800 °C, the amine species disappeared, and the pyrrolic-N and pyridine/triazine nitrogen obviously decreased. The different doped N contents and N species should play an important role in determining the CO_2_ adsorption and selectivity. The C1s XPS spectra of NPC-700-x and NPC-y-4 are displayed in Figure S3 and Figure 3. All the samples show similar C species, the C1s spectra of three peaks at 284.8, 286.1, and 289.9 eV can be relative to C-C/C=C, C=N/C-N/C-O and O=C-O, respectively [40,41]. This means that some -COOH and -OH can be preserved in the resultant NPCs.

**Figure 4:**
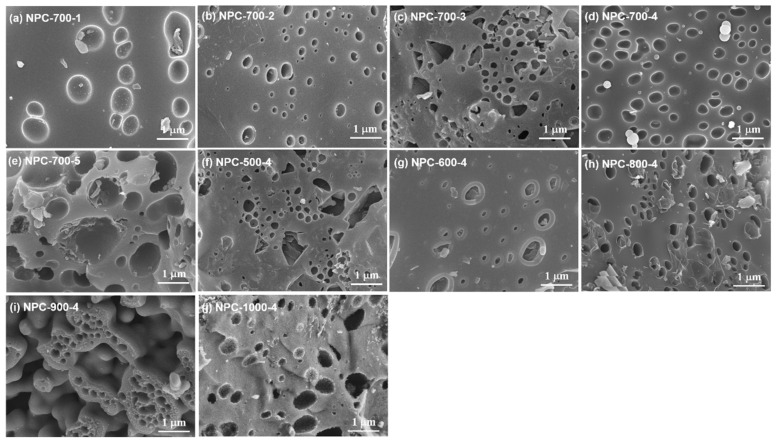
SEM images of NPC-700-x (**a**–**e**) and NPC-y-4 (**f**–**j**).

**Figure 5:**
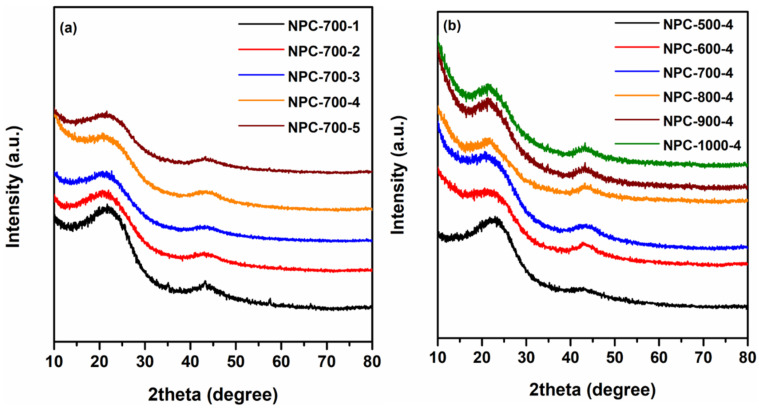
X-ray diffraction patterns of NPC-700-x (**a**) and NPC-y-4 (**b**).

Figure 4 shows the SEM morphology and structures of NPC-700-x and NPC-y-4. All the NPCs samples demonstrate an irregular shape with some obvious pores/cavities on the surface, confirming the pore-forming ability of KOH chemical activation [14]. The X-ray diffraction patterns of NPC-700-x and NPC-y-4 are displayed in Figure 5. All the NPC-700-x and NPC-y-4 samples demonstrate two weak broad diffraction peaks near 23° and 43°, corresponding to the (002) and (100) plane, respectively [34]. These weak peaks indicate the amorphous structures of the NPCs. Additionally, the increased intensities of peak (100) at 43° imply the presence of graphitized carbon and a higher degree of graphitization with the increasing activation temperature [42].

**Figure 6:**
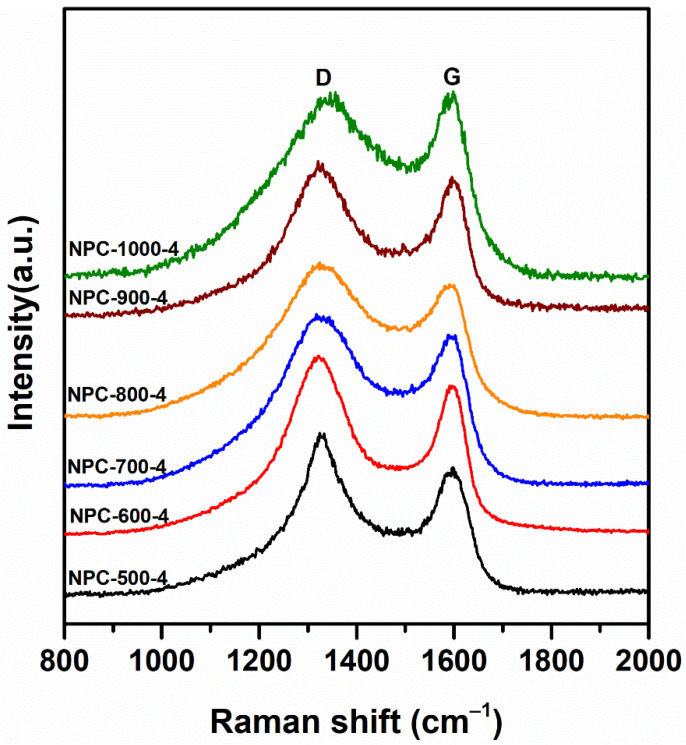
Raman spectra of NPC-y-4.

Figure 6 and Figure S4 display the Raman spectra of NPC-700-x and NPC-y-4. Two characteristic peaks at around 1596 cm^−1^ (G band) and 1320 cm^−1^ (D band) are associated with the E2g model of the graphite layer and the vibrations of carbons with dangling bonds, respectively [42]. The intensity ratio of the D band and G band (I_D_/I_G_) is indicative of the defects and disorder degree of the carbon materials. The I_D_/I_G_ values of NPC-700-x and NPC-y-4 exceed or reach 1.0 (Table S1), suggesting the amorphous carbon structure with a high content of lattice edges or defects [43]. Additionally, the I_D_/I_G_ ratios of NPC-y-4 decrease with the activation temperature (from 1.23 to 0.97), showing that a higher activation temperature can promote the degree of graphitization of the NPCs.

### 3.2. Textural Properties

**Figure 7:**
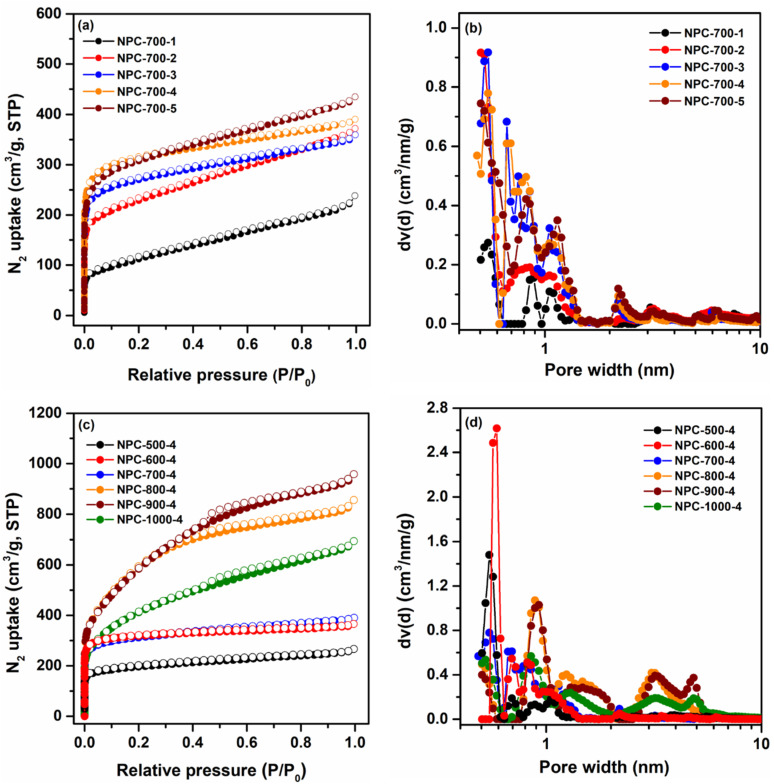
77 K N_2_ adsorption (filled) and desorption (empty) isotherms (**a**,**c**) and pore size distributions (**b**,**d**) of NPC-700-x and NPC-y-4.

The textural properties of the NPCs and RSS were investigated by 77 K N_2_ analysis (Figure 7 and Figure S5), and the derived specific surface areas, pore volumes, and porosity data were summarized in Table 2. The RSS shows the characteristic type-IV adsorption isotherms [44,45], indicating its mesoporous structure. The BET specific surface area (*S_BET_*) and total pore volume (*V_total_*) of RSS are only 40 m^2^/g and 0.049 cm^3^/g. As shown in Figure 7a,c, all the NPC-700-x and NPC-y-4 samples demonstrate a steep N_2_ uptake increase at a very low relative pressure region (P/P_0_ < 0.01), which is indicative of the abundant micropores in these resultant N-doped carbons [44,45]. In addition, the gradual N_2_ uptake increase at the higher relative pressure region suggests the presence of some mesopores. Furthermore, the pore size distribution curves (Figure 7b,d) show that the as-obtained NPCs possess a large number of ultramicropores (<0.7 nm), implying that NPCs are promising for the adsorption of CO_2_ with a molecular kinetic diameter of 0.33 nm [11,25,46].

As can be seen from Figure 7a and Table 2, the increase in the melamine dosage can greatly improve the porosity of NPC-700-x. Among the NPC-700-*x* samples, NPC-700-4 possesses the largest *S_BET_* (1190 m^2^/g), *S_micro_* (1010 m^2^/g), *V_micro_* (0.411 cm^3^/g), *V_ultramicro_* (0.21 cm^3^/g), and micropore volume ratio *V_micro_/V_total_* (0.682). These apparent sharp increases in porosity are attributed to the introduction of melamine during pyrolysis (Figure 4a–e) [47,48]. In addition, the trend of increasing porosity is basically consistent with the order of the doped nitrogen content. However, the porosity of NPC-700-5 decreases at a higher melamine addition. Thus, the optimum mass ratio of RSS, KOH, and melamine is 1:2:0.8.

On the other hand, activation temperature also plays an important role in tuning the porosity and chemical properties of NPC-y-4. As the activation temperatures increase, the *S_micro_*, *V_micro_*, micropore volume ratio *V_micro_/V_total_*, and doped N content increase continuously. This is because the increased high temperatures favor KOH in etching the carbon skeletons to generate a porous network [14]. NPC-800-4 has the highest S_BET_ (2163 m^2^/g), *S_micro_* (1209 m^2^/g), *V_micro_* (0.544 cm^3^/g), and *V_total_* (1.323 cm^3^/g), far surpassing all previously reported RSS-based PCs [28,29,30,31,32,49]. However, as the activation temperatures surpass 800 °C, the adsorption isotherms show an obvious hysteresis loop, suggesting the formation of mesoporous pore structures. Notably, the doped nitrogen content and the ultramicropore volumes drastically decrease due to the higher activation temperature.

### 3.3. CO_2_ Adsorption and Selectivity

**Figure 8:**
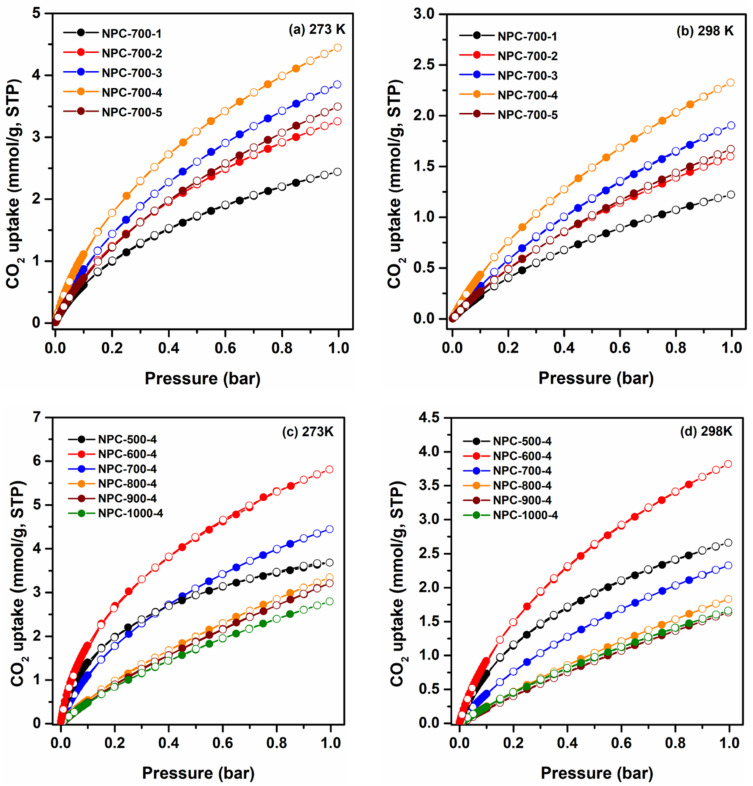
CO_2_ adsorption–desorption isotherms at 273 K and 298 K for NPC-700-x (**a**,**b**) and NPC-y-4 (**c**,**d**).

Motivated by both the high microporosity and the doped nitrogen content, the CO_2_ adsorption performance of the RSS-derived NPCs were investigated at 273 K and 298 K, respectively. Additionally, the corresponding CO_2_ adsorption–desorption isotherms at both temperatures are displayed in Figure 8. All the NPCs samples demonstrate a continuous increase in CO_2_ uptakes with the increasing pressure and have not yet reached saturation, suggesting that larger CO_2_ uptakes can be achieved at higher pressures [17,50]. Additionally, these completely reversible adsorption and desorption isotherms also confirm that the CO_2_ adsorption of the NPCs is physisorption in nature. Additionally, this is also evidenced by the obviously decreased CO_2_ uptakes at a higher temperature of 298 K (Figure 8b,d). Among the NPC-700-x, NPC-700-4 has the highest CO_2_ uptake of 4.45 mmol/g (273 K, 1.0 bar, Table 3), which is due to its large microporosity and high doped N content. The abundant polar N sites (amine, pyrrole N, and pyridine N) on the wall of micropores can strongly enhance the interaction between CO_2_ and the NPCs through quadrupole–dipole interaction. Interestingly, the CO_2_ adsorption performance of NPC-700-3 surpasses NPC-700-5. The doped N content, *S_BET_*, *S_micro_*, and *V_micro_* values of NPC-700-5 are even slightly higher than those of NPC-700-3, which should be attributed to its higher ultramicropore volume (Table 2). Additionally, the ultramicropores (<0.7 nm) are conducive to a greatly improved CO_2_ adsorption capacity and selectivity at low partial pressure [11,25].

NPC-600-4 shows much higher CO_2_ uptakes than other NPC-y-4 and NPC-700-x; the maximum CO_2_ uptakes can reach 5.81 mmol/g and 3.82 mmol/g at 1.0 bar, 273 K and 298 K (Table 3), respectively. This is mainly due to its simultaneous high doped nitrogen content (6.60 wt%), second largest *S_micro_* (1144 m^2^/g), the largest *V_micro_* (0.452 cm^3^/g), and the micropore volume ratio. In particular, the CO_2_ uptakes of NPC-600-4 can reach 2.29 mmol/g and 1.23 mmol/g at 0.15 bar, which is a typical CO_2_ partial pressure of flue gas. These values surpass some typical solid sorbents under identical measurement conditions, such as MOFs [7], covalent organic framework [10], zeolites [8] and N-rich porous organic polymer [51]. Interestingly, NPC-500-4 has the second largest CO_2_ uptake at low pressure (P < 0.4 bar, 273 K and P < 1 bar, 298 K), which is due to its higher amine content and large ultramicropore volume. The amine group and ultramicropores can significantly improve the CO_2_ adsorption capacity via molecular sieving and quadrupole–dipole interaction.

**Figure 9:**
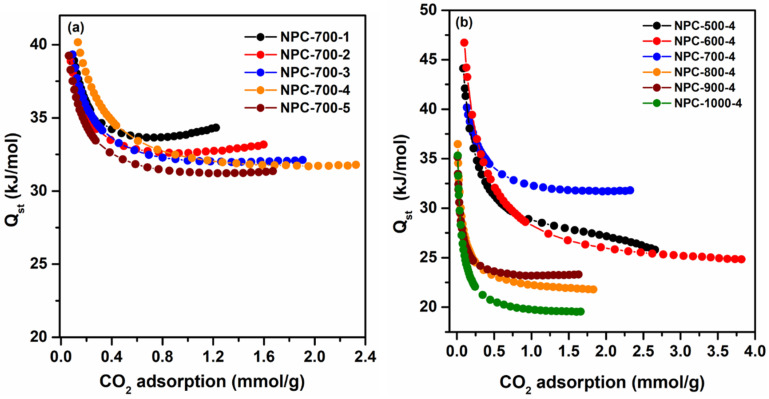
Isosteric heat of CO_2_ adsorption as a function of CO_2_ uptake for NPC-700-x (**a**) and NPC-y-4 (**b**).

To further understand the interaction between the CO_2_ molecules and the NPCs, the isosteric heat of adsorption (Q_st_) was calculated from the obtained adsorption isotherms (273 K, 298 K) in terms of the Clausius–Clapeyron equation [52]:

$$\ln P=\frac{Q_{\mathrm{st}}}{\mathrm{RT}}+C$$

where P is the pressure, Q_st_ (kJ/mol) is the isosteric heat of adsorption, R is the gas constant, T (K) is the temperature, and C is the equation constant. The dependencies of the Q_st_ and CO_2_ adsorption capacity are shown in Figure 9. For each sample, the Q_st_ values greatly decrease with the CO_2_ uptake, suggesting that the interaction between the CO_2_ molecules and the porous N-doped carbon surface is much stronger than that between the CO_2_ molecules [50,51]. Moreover, the ranking order of the Q_st_ values is basically consistent with the order of the CO_2_ uptakes. It can be observed that the Q_st_ values of NPC-700-x, NPC-500-4, and NPC-600-4 surpass 40 kJ/mol, implying that the interaction intensity is much stronger. This is mainly attributed to the higher basicity resulting from these basic N species, which can provide lone-pair electrons of N atoms. These polar N sites can promote CO_2_ affinity through dipole–quadrupole interaction. Among all the NPCs, NPC-600-4 has the highest Q_st_ values.

The limiting adsorption enthalpies of the NPCs at zero surface coverage (Q_0_) were also calculated from the CO_2_ adsorption isotherms at different temperatures, using the Virial equation and the Vant Hoff equation [53]. The plotted Viral curves and the k_0_, A_0_ data for NPC-700-x and NPC-y-4 are shown in Figure S6 and Table S2. The calculated Q_0_ values of NPC-700-x and NPC-y-4 are in the range of 33.9–36.4 kJ/mol and 21.8–37.4 kJ/mol (Table 3), respectively. The apparent Q_0_ decrease with the activation temperature increase should be ascribed to the loss of nitrogens at a higher activation temperature. These further evidence the importance of N doping on enhancing the CO_2_ adsorption of NPCs. Thus, for efficient low-pressure CO_2_ capture, a moderate activation temperature (<700 °C) should be adopted.

**Figure 10:**
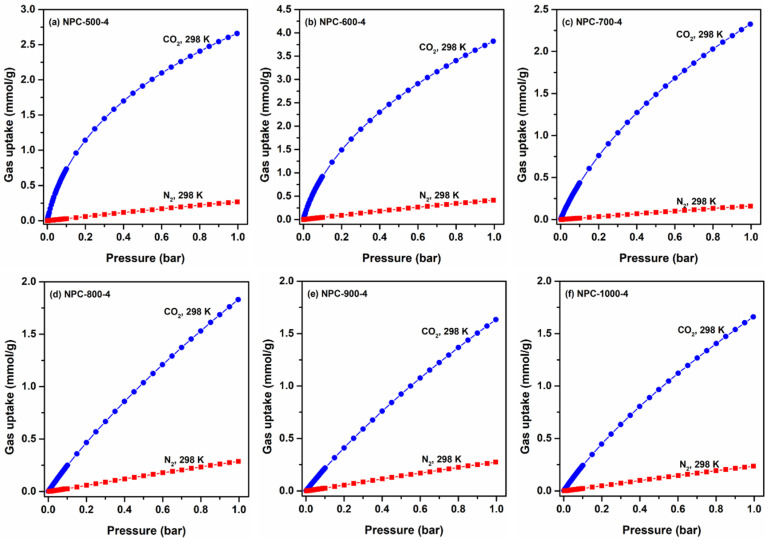
CO_2_ and N_2_ adsorption isotherms of NPC-y-4 (**a**–**f**) at 298 K.

**Figure 11:**
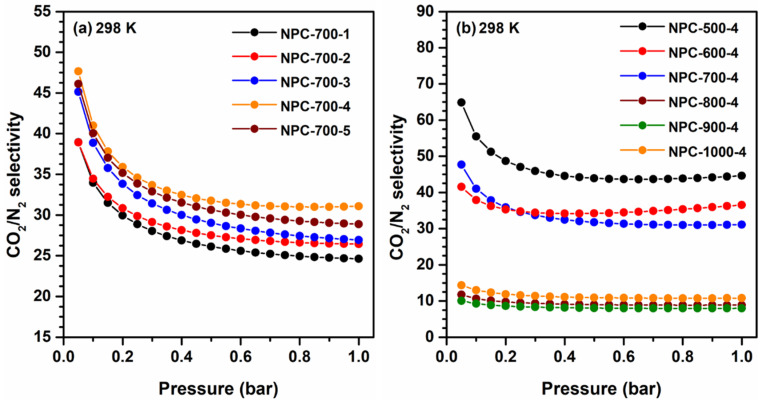
IAST CO_2_/N_2_ adsorption selectivity of NPC-700-x (**a**) and NPC-y-4 (**b**) at 298 K.

In order to evaluate the practical separation property of the NPCs, the adsorption isotherms of N_2_ at 298 K were measured and compared with those of CO_2_ in Figure S7 and Figure 10. It can be seen that the CO_2_ uptakes of all the NPCs samples are considerably larger than the N_2_ in the whole measured pressure range, indicating the high CO_2_/N_2_ adsorption selectivity. The ideal solution adsorption solution (IAST) was adopted to calculate the CO_2_/N_2_ adsorption selectivity from the simulated flue (15% CO_2_/85% N_2_) at 298 K. Figure 11 displays that the IAST CO_2_/N_2_ selectivities of the NPCs drop with the pressure increase. At 298 K and 1.0 bar, NPC-500-4 also exhibits a highest CO_2_/N_2_ selectivity of 44.6 (Table 3), resulting from its large ultramicropore volume and abundant doped nitrogens (amine, pyrrolic-N, pyridine-N, and graphitic-N). Compared with the N_2_ molecules, the CO_2_ molecules have a smaller molecular kinetic diameter and a larger quadrupole moment. These ultramicropores and polar N-containing sites can strongly improve the interaction between the CO_2_ molecules and the pore surface via the molecular sieving effect and the quadrupole–dipole interaction. NPC-800-4 possesses the largest micropore volume and BET surface area, while the CO_2_/N_2_ selectivity is only 10.8. This is mainly due to the low doped N content, confirming that higher polar nitrogen doping is the key factor for improving CO_2_/N_2_ selectivity. The CO_2_/N_2_ selectivity of NPC-600-4 is high up to 36.6, surpassing a large number of N-doped porous carbons under identical measurement conditions (Table 4) [17,18,54,55,56,57,58,59,60,61,62]. The high CO_2_/N_2_ adsorption selectivity and large CO_2_ uptakes under ambient conditions can be attributed to the ultramicroporosity and abundant doped N species.

**Figure 12:**
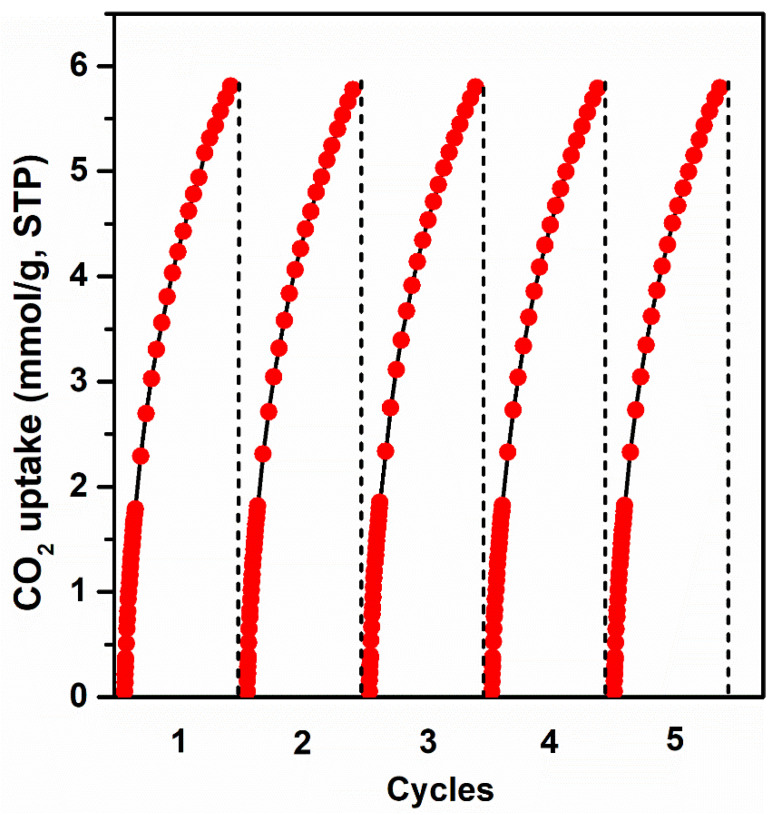
Five consecutive adsorption–desorption cycles of NPC-600-4 at 273 K.

In addition to the high CO_2_ adsorption capacity and the CO_2_/N_2_ selectivity, the recycle performance of the adsorbents also matters in practical applications. Figure 12 presents the five consecutive CO_2_ adsorption–desorption cycles of NPC-600-4 at 273 K. After each adsorption process, the adsorbent is regenerated by high-vacuum desorption, and it is directly used for another adsorption cycle. After five cycles, the CO_2_ adsorption capacity of NPC-600-4 merely drops, suggesting the excellent recycle performance. Given the superior CO_2_ adsorption capacity, CO_2_/N_2_ selectivity, and good recycle performance, NPC-600-4 is promising in CO_2_ capture applications.

## 4. Conclusions

In summary, a series of rubber seed shell-based N-doped porous carbons were prepared by one-pot high-temperature activation. The obtained NPCs demonstrated tunable microporosity and doped nitrogen content by adjusting the nitrogen source dosage and the activation temperature. The BET surface areas and doped nitrogen contents of the NPCs were in the range of 398–2163 m^2^/g and 0.82–7.52 wt%, respectively. It was found that the ultramicroporosity and polar nitrogens significantly affected the CO_2_ adsorption performances at low pressure. Among the RSS-based NPCs, highly microporous NPC-600-4 possesses the largest CO_2_ uptakes of 5.81 mmmol/g (273 K,1.0 bar) and 3.82 mmol/g (298 K, 1.0 bar), as well as the high CO_2_/N_2_ selectivity of 36.6, far exceeding a variety of reported biomass-based porous carbons. In addition, NPC-600-4 also shows excellent thermal stability and recycle performance, confirming the competitive application potential in practical CO_2_ capture.

## Figures and Tables

**Table 1:** Elemental analysis data for RSS and NPCs.

Samples	EA (wt%)				XPS ^b^ (wt%)		
	C	N	H	O ^a^	C	N	O
**RSS**	51.36	0.26	6.29	57.91	--	--	--
**NPC-700-1**	77.43	5.21	2.82	14.54	78.59	6.32	9.02
**NPC-700-2**	76.97	6.65	2.76	13.62	78.37	6.56	8.95
**NPC-700-3**	76.08	7.06	2.84	14.02	76.44	6.73	10.33
**NPC-700-4**	75.71	7.36	1.87	15.06	75.07	8.54	9.59
**NPC-700-5**	74.04	7.52	2.67	15.77	70.03	11.93	10.33
**NPC-500-4**	68.72	5.33	3.60	22.35	66.76	5.42	19.99
**NPC-600-4**	72.84	6.60	4.39	16.17	70.23	7.28	14.97
**NPC-800-4**	84.27	1.58	1.10	13.05	84.22	1.59	9.49
**NPC-900-4**	85.99	1.17	1.06	11.78	86.27	1.33	8.21
**NPC-1000-4**	87.32	0.82	1.64	10.22	92.29	0.92	4.26

^a^ The element O content was obtained by taking the difference between the total element content and the CHN element content. ^b^ The element C, N, and O contents (wt%) were calculated from the obtained XPS elemental atomic data for better comparison with the EA results.

**Table 2:** Textural parameters deduced from the 77 K N_2_ isotherms for RSS and NPCs.

Samples	*S_BET_*^a^ (m^2^/g)	*S_micro_*^b^ (m^2^/g)	*V_micro_*^b^ (cm^3^/g)	*V_ultramicro_*^c^ (cm^3^/g)	*V_total_*^d^ (cm^3^/g)	*V_micro_/V_total_*
**RSS**	40	0	0	0	0.049	0
**NPC-700–1**	398	114	0.053	0.043	0.369	0.144
**NPC-700-2**	823	436	0.187	0.135	0.575	0.325
**NPC-700-3**	1017	792	0.323	0.158	0.555	0.582
**NPC-700-4**	1190	1010	0.411	0.210	0.603	0.682
**NPC-700-5**	1139	828	0.348	0.148	0.674	0.516
**NPC-500-4**	734	551	0.226	0.285	0.411	0.550
**NPC-600-4**	1246	1144	0.452	0.179	0.565	0.800
**NPC-800-4**	2163	1209	0.544	0.061	1.323	0.411
**NPC-900-4**	2152	844	0.386	0.052	1.482	0.260
**NPC-1000-4**	1494	680	0.301	0.071	1.037	0.290

^a^ Specific surface area was calculated using the BET method. ^b^ Micropore specific surface area and micropore volume calculated using t-plot method. ^c^ Pore volume of ultramicropore (d < 0.7 nm) calculated using QSDFT method. ^d^ Total pore volume at *p*/*p*_0_ = 0.995.

**Table 3:** CO_2_ adsorption capacities, limiting adsorption enthalpies at zero coverage, and CO_2_/N_2_ selectivities of NPC-700-x and NPC-y-4.

Sample	CO_2_ Uptake (mmol/g)		Q_0_ ^a^ (kJ/mol)	IAST Selectivity ^b^
	273 K	298 K		CO_2_/N_2_
NPC-700-1	2.44	1.22	33.9	24.6
NPC-700-2	3.25	1.60	34.1	26.4
NPC-700-3	3.85	1.90	34.3	26.9
NPC-700-4	4.45	2.33	36.4	31.1
NPC-700-5	3.69	2.66	34.2	28.9
NPC-500-4	3.49	1.67	36.6	44.6
NPC-600-4	5.81	3.82	37.4	36.6
NPC-800-4	3.85	1.90	26.1	10.8
NPC-900-4	3.21	1.63	25.0	8.9
NPC-1000-4	2.80	1.66	21.8	8.0

^a^ The limiting enthalpy of adsorption at zero coverage (Q_0_) in kJ/mol. ^b^ Selectivity was calculated using the IAST method at a mole ration of 15:85 for CO_2_/N_2_.

**Table 4:** Comparison of CO_2_ adsorption values and CO_2_/N_2_ selectivity from reported N-doped porous carbons.

Samples	S_BET_ (m^2^ g^−1^)	CO_2_ Uptakes (mmol g^−1^)		IAST CO_2_/N_2_ Selectivity at 298 K	Ref.
		273 K/1.0 bar	298 K/1.0 bar		
**NPC-600-4**	1246	5.81	3.82	36.6	This work
**LS-600-0.3**	1188	5.11	3.68	16	[23]
**HS-500-3**	1600	6.43	4.30	17	[17]
**CN-600-3**	1082	5.12	3.71	13	[62]
**UC-650-2**	1394	6.27	4.40	17	[57]
**NDPC-10%-3**	1153	5.55	3.34	20.8 (273 K)	[59]
**NPC-2**	1384	5.86	-	18 (273 K)	[61]
**ACBK3**	1377	7.0	5.20	32.3	[60]
**WSM-550-2**	1535	5.86	4.32	19	[18]
**UC-15-2-600**	1113	-	4.80	22	[58]
**COL-900**	1382	4.41	2.88	47	[34]
**CN6-750-KOH**	1928	5.57	3.91	15	[56]
**PC-SK-2-3-800**	1418	5.61	3.82	13	[54]
**LCM-550-2**	1487	5.44	3.87	20	[55]

## Data Availability

Not applicable.

## Supplementary Materials

The following supporting information can be downloaded at https://www.mdpi.com/article/10.3390/nano12111889/s1, Figure S1: N1s XPS spectra of NPC-900-4 and NPC-1000-4, Figure S2: XPS (a) and N1s XPS (b–f) spectra of NPC-700-x, Figure S3: C1s XPS spectra of NPC-700-x, Figure S4: Raman spectra of NPC-700-x, Figure S5: 77 K N_2_ adsorption–desorption isotherms (a) and pore size distribution (b) of RSS, Figure S6: Virial plots of NPC-700-x and NPC-y-4, Figure S7: CO_2_ and N_2_ adsorption isotherms of NPC-700-*x* and NPC-*y*-4 at 298 K, Table S1: I_D_/I_G_ values of NPC-y-4, Table S2: KH, A_0_, and Q_0_ values of CO_2_ adsorption in the NPC-700-x and NPC-y-4.
