# Supplementary material for: One-Pot Synthesis of Rubber Seed Shell-Derived N-Doped Ultramicroporous Carbons for Efficient CO2 Adsorption
Source: Nanomaterials (Basel). 2022 May 31;12(11):1889. doi: 10.3390/nano12111889 (PMC9182511; doi:10.3390/nano12111889)
Supplement: Supplementary file 1 [file nanomaterials-12-01889-s001.zip › nanomaterials-1725518-supplementary.pdf]

One-pot synthesis of rubber seed shell-based N-doped ultramicroporous carbons for efficient CO_2 adsorption

Xiaoxia Zhang [1], Meng Rong [2], Hui Cao [1,*] and Tianwei Tan [1,*]

[1] Beijing Key Laboratory of Bioprocess, College of Life Science and Technology, Beijing University of Chemical Technology, Beijing 10029, China; zhangxiaoxia0416@163.com

[2] CAS Key Laboratory of Green Process and Engineering, Institute of Process Engineering, Chinese Academy of Sciences, Beijing 100190, China; mrong@ipe.ac.cn

* Correspondence: caohui@mail.buct.edu.cn (H.C.); twtan@mail.buct.edu.cn (T.T.)

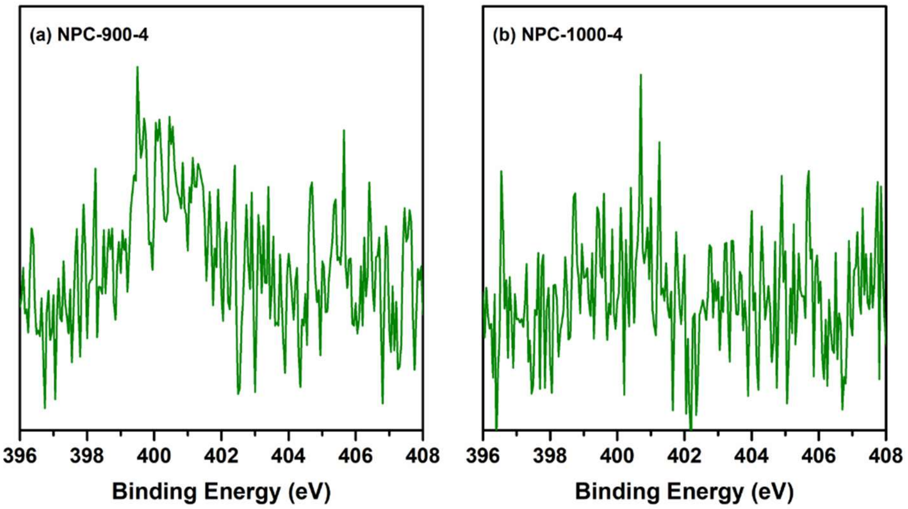

Figure S1. N1s XPS spectra of NPC-900-4 （a） and NPC-1000-4 （b）

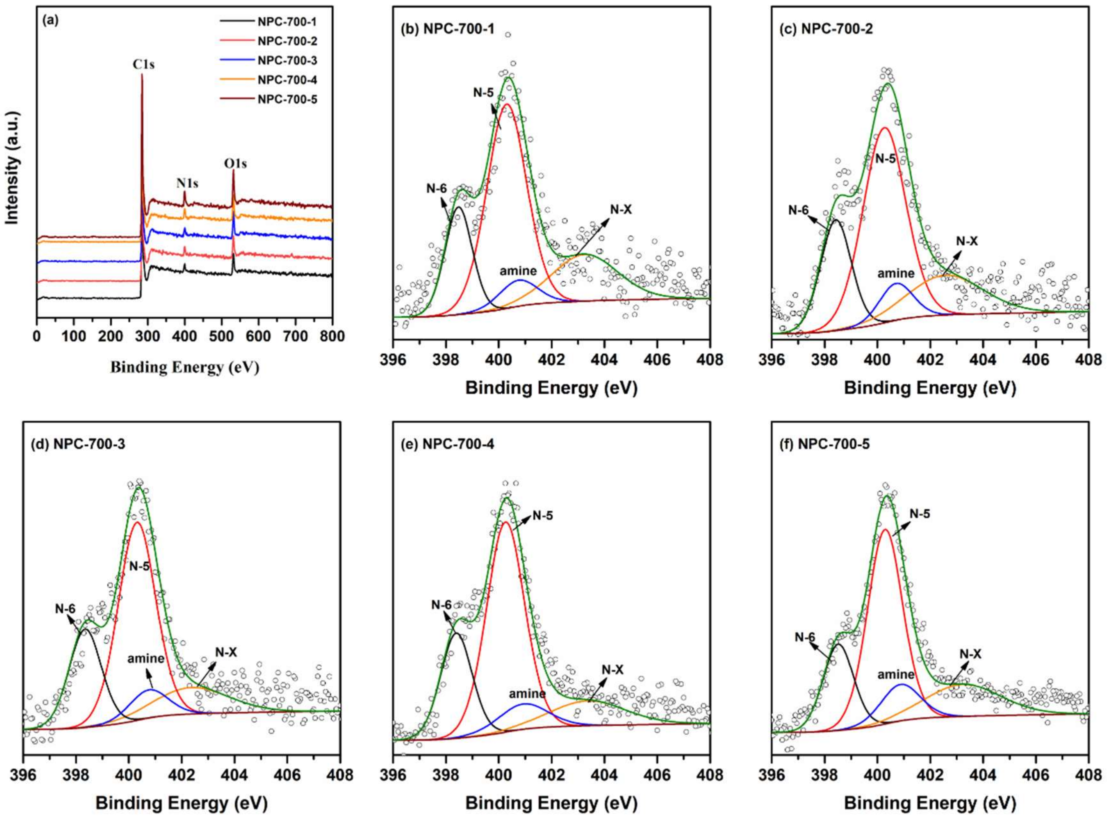

Figure S2. XPS (a) and N1s XPS (b-f) spectra of NPC-700-*x*.

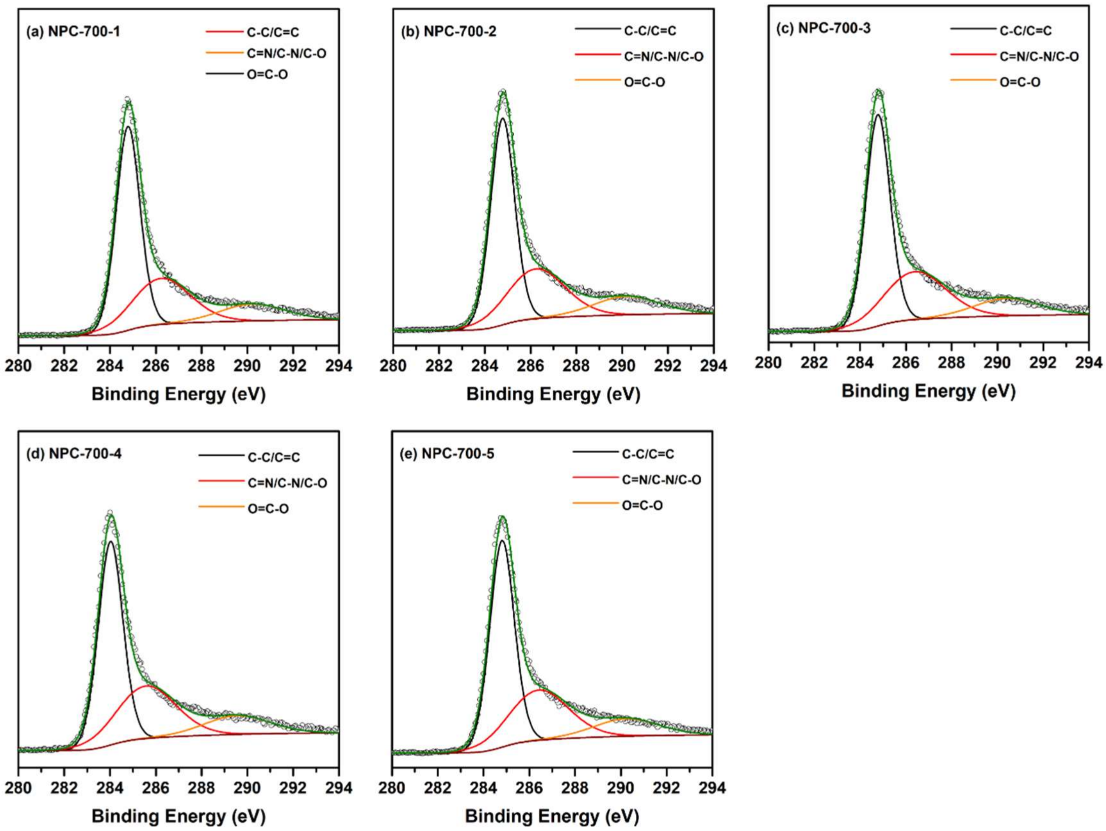

Figure S3. C1s XPS spectra of NPC-700-*x* （a-e）.

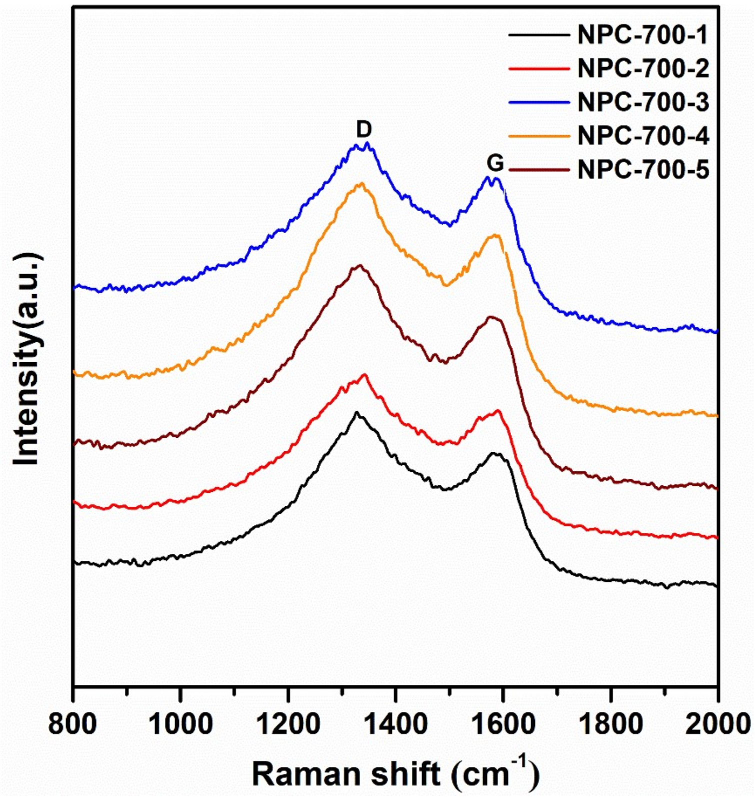

Figure S4. Raman spectra of NPC-700-*x*.

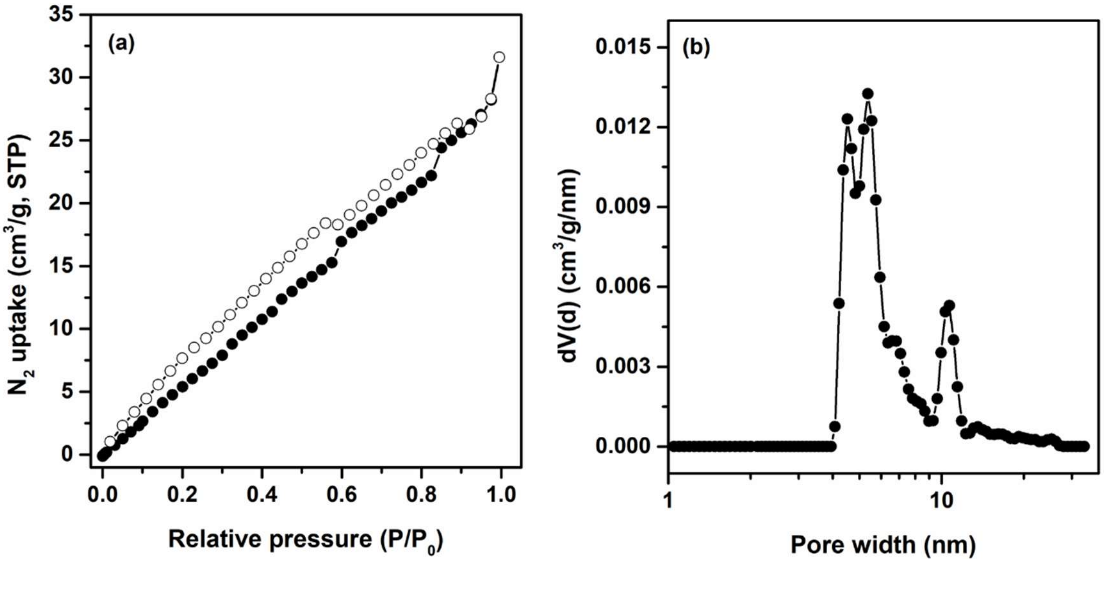

Figure S5. 77 K N_2 adsorption-desorption isotherms (a) and pore size distribution (b) of RSS.

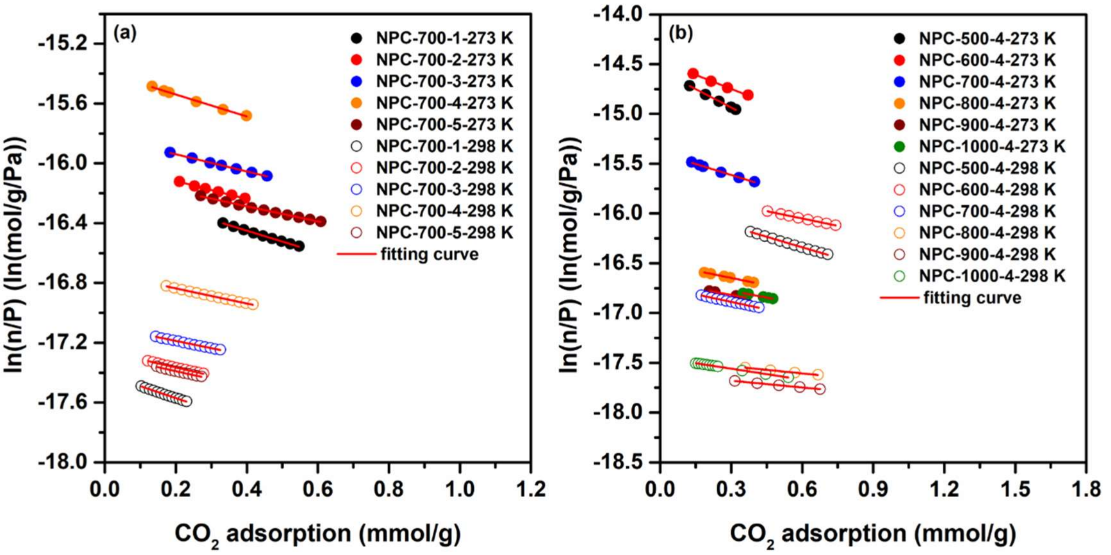

Figure S6. Virial plots of NPC-700-*x* （a） and NPC-*y*-4 （b）.

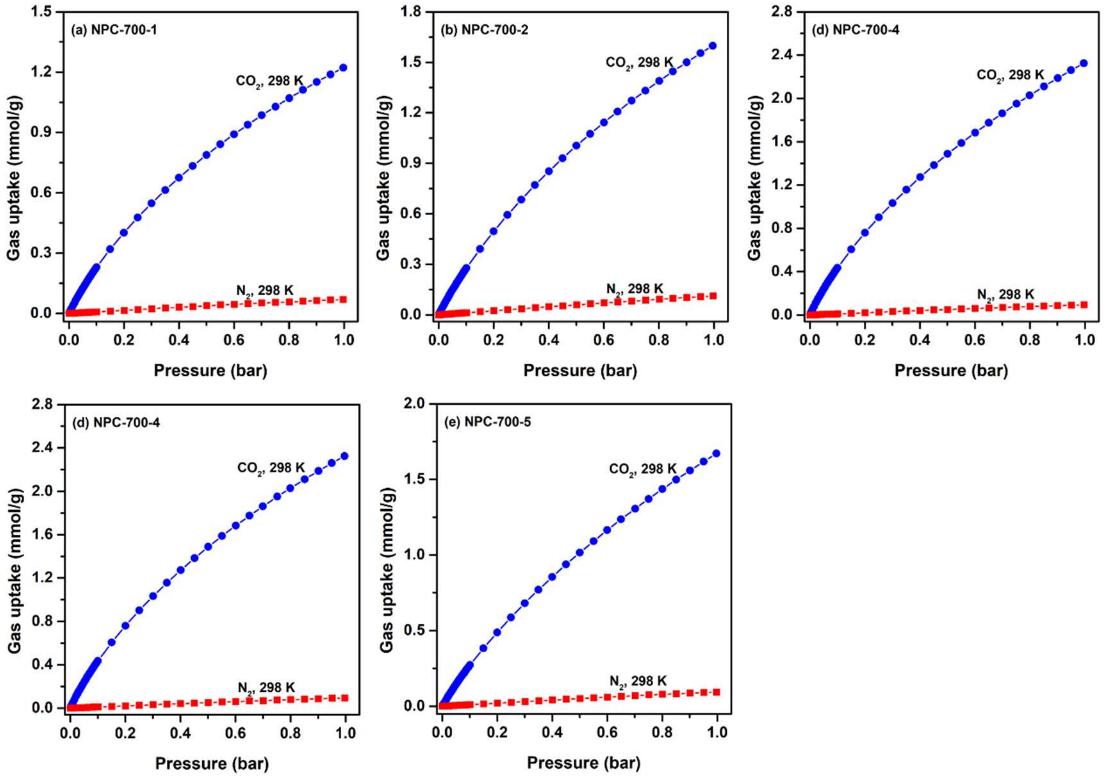

Figure S7. CO_2 and N_2 adsorption isotherms of NPC-700-*x* and NPC-*y*-4 at 298 K （a-e）.

Table S1 I_D/I_G values of NPC-y-4.

	NPC-500-4	NPC-600-4	NPC-700-4	NPC-800-4	NPC-900-4	NPC-1000-4
ID/IG	1.23	1.16	1.12	1.11	1.09	0.97

Table S2. K_H, A_0, and Q_0 values of CO_2 adsorption in the NPC-700-*x* and NPC-*y*-4.

Sample	*T/K*	K_H/mol/(g.Pa)	A_0/ln(mol/(g.Pa))	R^2	Q_0
NPC-700-1	273	-16.159	9.60123E-08	0.999	33.9
	298	-17.411	2.74511E-08	0.998	
NPC-700-2	273	-15.996	1.12978E-07	0.998	34.1
	298	-17.257	3.20317E-08	0.999	
NPC-700-3	273	-15.824	1.34215E-07	0.999	34.3
	298	-17.090	3.78381E-08	0.999	
NPC-700-4	273	-15.392	2.06642E-07	0.998	36.4
	298	-16.735	5.39575E-08	0.999	
NPC-700-5	273	-16.021	1.10197E-07	0.999	34.2
	298	-17.285	3.11276E-08	0.998	
NPC-500-4	273	-14.567	4.71767E-07	0.999	36.6
	298	-15.916	1.22344E-07	0.999	
NPC-600-4	273	-14.460	5.25056E-07	0.998	37.4
	298	-15.840	1.32061E-07	0.999	
NPC-800-4	273	-16.500	6.82335E-08	0.999	26.1
	298	-17.464	2.60325E-08	0.999	
NPC-900-4	273	-16.688	5.65472E-08	1.000	25.0
	298	-17.611	2.24622E-08	0.998	
NPC-100-4	273	-16.646	5.90017E-08	0.999	21.8
	298	-17.449	2.64243E-08	0.999	
